# Royal Statistical Society

**Published:** 1897-02-27

**Authors:** 


### Medical Progress and Hospital Clinics.

[*The Editor will be glad to receive offers of co-operation and contributions from members of the profession. All letters should be addressed to* The Editor, at the Office, 28 & 29, Southampton Street, Strand, London, W.C.]

### ROYAL STATISTICAL SOCIETY.

At a meeting held on Tuesday, the 16th instant, a paper was read by Mr. Noel A. Humphreys on "English Vaccination and Small-pox Statistics; with special reference to the Report of the Royal Commission, and to recent Small-pox Epidemics." The paper, in the main, consisted of an analysis of the evidence given before the recent Royal Commission, of the Commissioners' final report, and of the abundant statistics contained in that report. The author arrived at the following conclusions: (1) That the mean annual death-rate from small-pox at all ages in England and Wales, which was 408 per 1,000,000 in the twelve years for which records exist prior to compulsory vaccination, fell to 126 per 1,000,000 in the forty-two years since vaccination was made compulsory, which period included the world-wide epidemic of 1871-72. (2) That the main part of this decline of mortality occurred among children under the age of ten years, who may be presumed to have been principally affected by the increase of infant vaccination. (3) That the age-incidence of fatal small-pox had entirely changed under the influence of increased infant vaccination; and that small-pox, from being mainly a disease of childhood, was now proportionately more fatal to adults. (4) That, judged by the statistics of the last six principal local small-pox epidemics, the proportion of deaths of children bore a constant ratio to the proportion of children officially returned "unaccounted for" as regards successful vaccination. (5) That efficient infant vaccination conferred a practically complete immunity from fatal small-pox during the first ten years of life. (6) That the increase in the proportion of adult deaths from small-pox must be attributed to the waning of the protective effect of infant mortality; this hypothesis being corroborated by the steady increase in the proportional case-mortality of vaccinated persons in recent epidemics, at successive age-periods above ten years. (7) That both the small-pox attack rate and the case-mortality was far lower at all ages among those who had been vaccinated in infancy among the unvaccinated. (8) That the type of disease suffered by small-pox patients was far more severe among the unvaccinated than among the vaccinated; and that the severity of the type appeared to vary with the number and quality of the vaccination marks. (9) That, judged by the statistics of small-pox hospital nurses and attendants, successful re-vaccination afforded practically complete protection to adults, even to those brought into actual personal contact with the worst forms of disease.

